# Can the Stroop Test Be Useful in Differentiating Specific Learning Disorder from Attention Deficit Hyperactivity Disorder?

**DOI:** 10.1192/j.eurpsy.2025.449

**Published:** 2025-08-26

**Authors:** H. A. Güler, F. U. Dündar, M. Kuz, M. E. Tezcan

**Affiliations:** 1 Department of Child and Adolescent Psychiatry, Selçuk University; 2 Special Clinic, Konya, Türkiye

## Abstract

**Introduction:**

Specific Learning Disorder (SLD) is a significant comorbidity in children with Attention Deficit Hyperactivity Disorder (ADHD). Both ADHD and SLD are neurodevelopmental conditions that share common characteristics, such as deficits in executive functions. Identifying SLD in patients with ADHD is crucial because targeted educational interventions are the primary treatment for SLD.

**Objectives:**

This study aimed to evaluate the utility of the Stroop Test (ST) in differentiating SLD from ADHD.

**Methods:**

79 patients (42 with ADHD and 37 with both ADHD and SLD) participated in the study. Sociodemographic information and ST performance metrics (completion time, omission errors, and commission errors) were collected by a child and adolescent psychiatrist. Additionally, parents completed the Turgay DSM-IV ADHD Rating Scale to assess the severity of ADHD symptoms.

**Results:**

There were no significant differences between the ADHD and ADHD+SLD groups regarding age, gender, or ADHD symptom severity. The ADHD+SLD group exhibited longer completion times across all sections of the ST. Omission errors in the fourth and fifth sections were significantly higher in the ADHD+SLD group. After adjusting for age, gender, and ADHD symptom severity, the completion time in the fifth section and omission errors in the fourth and fifth sections remained significant. Receiver Operating Characteristic (ROC) analysis identified cut-off scores for the fifth section’s completion time (42 seconds; Sensitivity: 0.62, Specificity: 0.66) and omission errors (1 error; Sensitivity: 0.64, Specificity: 0.61). Moreover, omission errors in the fifth section predicted being in the ADHD+SLD group (p= .006, Odds Ratio [OR]= 1.527, 95% CI= 1.127–2.068).

**Conclusions:**

The findings suggest that the ST may be a valuable tool for diagnosing SLD in patients with ADHD. In particular, the completion time and omission errors in the fifth section of the ST may serve as practical diagnostic indicators. Further research with larger sample sizes is necessary to confirm these results.

**Disclosure of Interest:**

None Declared

